# Gastrodin alleviates mild cognitive impairment and Alzheimer’s disease via candidate targeting of ACHE, S1PR5, SERPINE1, and TOP2A: an integrative computational and cellular study

**DOI:** 10.3389/fphar.2026.1937359

**Published:** 2026-09-29

**Authors:** Yu Wang, Meng-xi Liu, Jia-lu Zhu, Gui-xian Li, Hong Yu, Cong-hui Wang, Qiang Zong, Wen-sheng Wang

**Affiliations:** 1 Innovation Institute of Integrated Traditional Chinese and Western Medicine, Institute of Brain Science and Brain-inspired Research, Shandong First Medical University & Shandong Academy of Medical Sciences, Jinan, China; 2 Taihe Hospital, Hubei University of Medicine, Shiyan, China; 3 Hubei Key Laboratory of Embryonic Stem Cell Research, Department of Human Anatomy, Hubei University of Medicine, Shiyan, China; 4 Traditional Chinese Medicine (TCM) Rehabilitation Department, Danjiangkou Liuliping Central Health Center, Shiyan, China; 5 Department of Neurosurgery, Shengli Oilfield Central Hospital, Dongying, China

**Keywords:** Alzheimer’s disease, Aβ oligomer, gastrodin, machine learning, mild cognitive impairment, molecular dynamics, network pharmacology, neuroprotection

## Abstract

**Background:**

Alzheimer’s disease (AD) and its prodromal mild cognitive impairment (MCI) lack effective disease-modifying therapeutic strategies. Gastrodin (GAS), the major bioactive compound of *Gastrodia elata*, exhibits pronounced neuroprotective effects. However, the precise candidate molecular targets and systematic mechanisms by which GAS protects against AD and MCI remain poorly clarified; target engagement is inferred from computational simulation rather than direct biochemical validation.

**Methods/results:**

This study adopted an integrated approach of network pharmacology, transcriptome-based machine learning, molecular simulation, and *in vitro* cellular validation to explore the therapeutic mechanisms of GAS against AD/MCI. A total of 127 overlapping therapeutic targets of GAS, AD, and MCI were identified, which were mainly enriched in synaptic transmission and calcium signaling pathways. Four core hub genes (*ACHE*, *S1PR5*, *SERPINE1*, and *TOP2A*) were further screened by intersecting candidate targets with AD differentially expressed genes from the GSE329677 dataset. The ensemble Logistic + SVM machine learning model exhibited optimal diagnostic efficiency with an AUC value of 0.897. Molecular docking and 50 ns molecular dynamics simulations confirmed that GAS stably bound to the four target proteins and maintained stable binding states under physiological conditions. In an Aβ_1-42_ oligomer-injured N2a neuronal model, GAS showed no significant cytotoxicity at concentrations ranging from 0.1 to 100 μM. Notably, 25 μM GAS markedly reversed Aβ_1-42_-induced neuronal death and excessive intracellular ROS accumulation.

**Conclusion:**

This study identifies four novel candidate core therapeutic targets of GAS against AD/MCI and provides computational evidence for their stable atomic-level binding characteristics. GAS exerts prominent neuroprotective effects through anti-apoptotic and antioxidant mechanisms in Aβ-injured neurons. These findings provide a novel theoretical basis and preclinical evidence for the development of GAS as a natural candidate agent for AD intervention.

## 1 Introduction

Mild cognitive impairment (MCI) represents the critical prodromal stage of Alzheimer’s disease (AD), characterized by mild episodic memory decline and subtle cognitive dysfunction without obvious impairment of daily living ability (Li et al., 2023; Swanson et al., 2021). Epidemiological evidence has demonstrated that approximately 10%–15% of MCI patients progress to overt AD annually, making this transitional period a pivotal and optimal intervention window to block or delay irreversible neurodegenerative progression (Zawar et al., 2024). As the most prevalent progressive neurodegenerative disorder, AD is pathologically characterized by extracellular β-amyloid (Aβ) aggregation and plaque deposition, intracellular neurofibrillary tangle formation, excessive neuronal oxidative stress, sustained neuroinflammation, and massive neuronal loss, ultimately leading to irreversible cognitive deterioration and neurological dysfunction (Zhang and Zhang, 2024). Currently, clinically available therapeutic agents for AD can alleviate superficial symptom relief but not reverse or halt disease progression, and the limited efficacy and potential side effects of synthetic drugs severely restrict their long-term clinical application (Turgutalp and Kizil, 2024). Therefore, exploring safe, efficient, and multitarget natural neuroprotective compounds has become a research hotspot for the prevention and intervention of AD and MCI.

*Gastrodia elata* Blume, a classic traditional Chinese medicinal herb, has been widely used for the clinical treatment of amnesia, dizziness, and neurological disorders for thousands of years (Gong et al., 2022). Gastrodin, the primary bioactive phenolic glycoside monomer extracted from *G. elata*, possesses multiple neuroprotective pharmacological properties, including anti-inflammatory, antioxidant, antiapoptotic and synaptic plasticity-regulating activities, and has been proven to effectively improve learning and memory deficits in multiple neurological disease models (Luo et al., 2022; Xiao et al., 2023). Our previous preclinical work clarified the neuroprotective and antineuroinflammatory effects of gastrodin in AD-related pathological processes. In prior studies, we demonstrated that gastrodin ameliorates neuroinflammation and microglial overactivation both *in vivo* and *in vitro* by regulating the TLR4/TRAF6/NF-κB signaling axis and STAT3 phosphorylation. In LPS-induced neuroinflammatory model mice and BV-2 microglia, gastrodin inhibited microglial M1 polarization, reduced the release of proinflammatory cytokines, suppressed excessive astrocyte activation, and thereby rescued learning and memory impairment. Furthermore, TRAF6 overexpression rescue experiments confirmed the essential regulatory role of the TLR4/TRAF6/NF-κB cascade in mediating the anti-inflammatory activity of gastrodin (Wang et al., 2024). These findings lay a solid preclinical foundation for the neuroprotective efficacy of gastrodin against AD-associated neuronal damage.

Nevertheless, existing studies have focused mainly on the regulation of inflammatory signaling by gastrodin, and the core therapeutic targets and systematic molecular mechanisms underlying gastrodin-mediated protection against AD and early-stage MCI remain insufficiently explored. To fill these research gaps, the present study adopted a multidimensional integrative strategy to further explore the core molecular mechanism and therapeutic basis of gastrodin in AD and MCI. This study focused on undiscovered core therapeutic targets and systematic regulatory networks of gastrodin and further supplemented the evidence of atomic-level drug binding and neuronal phenotypic verification. In brief, we identified four novel AD-associated core hub genes of gastrodin, validated its stable molecular binding with key target proteins, and confirmed the reliable antioxidative and antiapoptotic neuroprotective effects of gastrodin in Aβ-injured neuronal models. Collectively, the findings of this study substantially complement and improve the existing mechanistic system by which gastrodin protects against cognitive impairment. By constructing a more comprehensive multitarget regulatory network, this work provides novel molecular insights and solid preclinical support for interventions for AD and early MCI and further promotes the translational application of gastrodin as a promising natural neuroprotective agent for the prevention and treatment of neurodegenerative cognitive disorders.

## 2 Materials and methods

### 2.1 Network pharmacology analysis

Disease-related protein-coding genes associated with Alzheimer’s disease and mild cognitive impairment were retrieved from the GeneCards database via standardized keyword retrieval strategies, and redundant gene entries were eliminated to generate independent target sets for each disease. The simplified canonical molecular-input line-entry system structure of gastrodin was imported into both the SwissTargetPrediction and TargetNet online platforms for human protein target prediction (Zhang et al., 2023). Predicted targets with a probability score exceeding 0.5 from both databases were retained, and duplicate gene symbols were removed to compile the candidate target dataset of gastrodin. Intersection analysis of the gastrodin target set, the AD target set, and the MCI target set was performed via the VennDiagram package in R, and overlapping genes were defined as the shared therapeutic targets of gastrodin that act on AD and MCI.

Shared therapeutic targets were uploaded to the STRING database to obtain human protein‒protein interaction (PPI) pairs with a confidence cutoff >0.7. The raw interaction data were exported and visualized via Cytoscape 3.9.1 (Bisht et al., 2024). The cytoNCA plugin was adopted to compute topological indices (degree, betweenness centrality, and closeness centrality) of all nodes to preliminarily screen hub genes with core regulatory roles in the PPI network. The cytoNCA plugin was adopted to compute topological indices (degree, betweenness centrality, and closeness centrality) of all nodes in the PPI network. Genes satisfying the threshold that all three topological metrics ranked within the top 50% of all 127 nodes were retained as hub genes. Gene Ontology functional enrichment and Kyoto Encyclopedia of Genes and Genomes signaling pathway enrichment were conducted for all shared therapeutic targets via the clusterProfiler R package, with adjusted *P* values less than 0.05 defined as the significance cutoff for enriched entries. Gene Ontology enrichment results were categorized into biological process (BP), cellular component (CC), and molecular function (MF) subclasses, and bubble plots were generated to visualize significantly enriched functional modules and signaling pathways.

### 2.2 Transcriptome data mining and core gene screening

The transcriptomic dataset GSE329677 containing gene expression profiles of 15 Gy γ-irradiated senescent human skin-derived fibroblasts (*n* = 88) and control fibroblasts (*n* = 63) was downloaded from the Gene Expression Omnibus public database. Raw expression matrix normalization and differential gene screening were completed via the limma R package, with screening thresholds set as an absolute log2-fold change greater than 1 and an adjusted *P* value less than 0.05 (Chen et al., 2023). Venn overlap analysis was executed between the screened differentially expressed genes associated with AD and the shared therapeutic targets of gastrodin to extract intersecting core hub genes, and box plots were drawn to verify the significant differential expression patterns of these core genes between the AD and control sample cohorts.

The intersecting core genes were selected as feature variables for classification modeling to distinguish AD patients from healthy controls. Six independent machine learning algorithms, including logistic regression, extreme gradient boosting, support vector machine, random forest, k-nearest neighbor and naive Bayes, were adopted, alongside recursive partitioning classification (Crampon et al., 2022; Zhang et al., 2026). All pairwise algorithm combinations were integrated to construct ensemble classification models, and area under the receiver operating characteristic curve values were calculated to evaluate the classification performance of individual core genes and each combined ensemble model (Zhu et al., 2026). A heatmap was generated to summarize the area under the curve values of all pairwise algorithm combinations, and receiver operating characteristic curves of the optimal performing ensemble models and single core genes were visualized for comparative analysis.

### 2.3 Molecular docking

Crystal three-dimensional structures of the ACHE, S1PR5, SERPINE1 and TOP2A proteins were downloaded from the RCSB Protein Data Bank database. PyMOL software was used to remove bound water molecules and redundant peptide chains from each protein crystal structure, and AutoDockTools 1.5.7 was applied to add hydrogen atoms and balance the molecular charge distribution for protein preparation (Majed et al., 2026). The two-dimensional chemical structure of gastrodin was obtained from the PubChem database, and molecular conformation energy minimization was performed under the MMFF94 force field before the optimized ligand structure was exported in PDBQT format. The grid box parameters were adjusted to fully cover the native ligand binding pocket of each target protein, and semiflexible molecular docking calculations were executed via AutoDock Vina software (Bisht et al., 2024). The docking conformation corresponding to the lowest binding free energy value was selected as the optimal binding state of gastrodin with each target protein. PyMOL and Discovery Studio software were jointly utilized to analyze intermolecular interaction modes, label hydrogen bond distances and identify key amino acid residues participating in ligand‒protein binding. For re-docking validation, crystal structures with PDB IDs 7D9O, 7EW1, 3CVM, and 1ZXM were tested using identical docking parameters. Co-crystallized ligands were extracted and re-docked back into their respective apo-protein binding pockets; a heavy-atom root-mean-square deviation (RMSD) < 2.0 Å between the re-docked pose and the original co-crystallized ligand was defined as the acceptable threshold (Alnajeebi, 2026).

### 2.4 Molecular dynamics simulation

Molecular dynamics (MD) simulation and MM/GBSA binding free energy analysis were performed via AMBER 24 to assess the structural stability and binding affinity of the gastrodin–target protein complexes (Zurmühl et al., 2026). Using docked complex structures as initial models, gastrodin was parameterized with AM1-BCC charges and the GAFF2 force field, whereas target proteins were described by the ff14SB force field. All systems were solvated in octahedral TIP3P water boxes (10 Å buffer) and charge-neutralized with Na/Cl ions. After gradient energy minimization, the systems were heated, equilibrated in NVT and NPT ensembles, and finally subjected to a 50 ns NPT production simulation. The simulations adopted a 10 Å nonbonded cutoff, PME electrostatics, SHAKE bond constraint, and Langevin temperature control (298.15 K, 2 ps) (Kumar et al., 2026). Simulation equilibration was defined as protein-backbone RMSD and complex Rg fluctuating within 0.1 nm without persistent upward drift after the initial 10 ns production run. Trajectories were sampled every 10 ps, and only the final 10-ns (40–50 ns) equilibrated trajectories were extracted for MM/GBSA calculation to discard unstable early-relaxation conformations and mitigate computational noise. The solvation free energy was computed via the igb = 2 GB model and surface-area-based nonpolar term, whereas the conformational entropy was omitted to reduce the computational cost. Therefore, the obtained free energy values are only suitable for comparing relative binding affinities among different complexes and should not be interpreted as absolute binding free energies. Free energy landscape (FEL) was constructed using RMSD and radius of gyration (Rg) as reaction coordinates rather than principal components derived from PCA, to characterize the conformational free energy minima of gastrodin–target complexes.

### 2.5 *In vitro* verification of cellular function

#### 2.5.1 Cell line, primary reagents and preparation of the Aβ_1-42_ oligomer

The mouse N2a (Neuro-2a mouse neuroblastoma cells) neuronal cell line was purchased from Tai’an Jiaheng Biotechnology Co., Ltd. Gastrodin (purity ≥98%, HPLC grade, Cat. No. G828303) was obtained from Shanghai Macklin Biochemical Co., Ltd. Fetal bovine serum and high-glucose DMEM were obtained from Servicebio. Penicillin--streptomycin solution, a CCK-8 assay kit, propidium iodide (PI) staining solution, and a DCFH-DA ROS fluorescent probe were purchased from Biosharp. Synthetic Aβ peptide (Cat. No. AG912) was acquired from Sigma-Aldrich.

For Aβ oligomer preparation, lyophilized Aβ_1-42_ powder was fully dissolved in ice-cold hexafluoroisopropanol to eliminate preformed aggregates, followed by vacuum drying at room temperature. The dried peptide was reconstituted in dimethyl sulfoxide, diluted to a final concentration of 100 μM with serum-free DMEM, and then incubated at 4 °C for 24 h to form a mature Aβ oligomer solution (Kadamangudi et al., 2024). After oligomerization incubation at 4 °C for 24 h, the 100 μM stock oligomer solution was further diluted with serum-free DMEM to obtain a 2 μM working solution immediately before cell treatment.

#### 2.5.2 Cell culture and grouping treatment

N2a cells were maintained in complete DMEM supplemented with 10% fetal bovine serum and 1% penicillin‒streptomycin and incubated under constant conditions of 37 °C and 5% CO_2_ saturated humidity. The culture medium was changed every 48 h, and the cells were passaged via 0.25% trypsin digestion when the cell confluence reached 80%.

For the cytotoxicity screening experiments, the cells were seeded into 96-well plates and treated with 0 μM, 0.1 μM, 1 μM, 10 μM, 50 μM, or 100 μM gastrodin for 24 h before viability was detected. For neuroprotective functional assays, cell cultures were divided into four independent experimental groups with three biological replicates per group. The control group received routine culture with complete medium without any additional treatment. The Model group was incubated with 2 μM Aβ oligomers to induce neuronal injury. The 5 μM and 25 μM gastrodin intervention groups were simultaneously treated with the corresponding concentrations of gastrodin and 2 μM Aβ_1-42_ oligomer (Wang et al., 2024). All the groups were continuously cultured for another 24 h after administration to establish the model and for drug intervention.

#### 2.5.3 CCK-8 cell viability assay

The cells were seeded evenly into 96-well culture plates at a uniform density, and the corresponding gradient gastrodin treatments were maintained for 24 h as designed (Luo et al., 2020). Ten microliters of CCK-8 working reagent was added to each well, and the plates were incubated in a 37 °C incubator for 2 h without light exposure. The absorbance values at 450 nm were measured via a microplate reader, and the relative cell viability of each concentration group was calculated by normalizing the absorbance to that of the untreated control group.

#### 2.5.4 PI staining

Adherent cells on coverslips were directly fixed and permeabilized, stained with PI working solution for 15 min at room temperature in the dark, sealed, and imaged under an inverted fluorescence microscope to quantify neuronal death, and the staining procedure was carried out in accordance with previously reported methods (Watts, 2021).

#### 2.5.5 Intracellular ROS detection

DCFH-DA fluorescent probe stock solutions were diluted with serum-free medium to prepare working solutions at a final concentration of 10 μM. The culture medium was discarded from the cell slides of each group, and the diluted probe mixture was added for incubation at 37 °C for 20 min. After thorough washing with phosphate-buffered saline to remove unbound probe, the slides were observed and photographed under a confocal laser microscope to compare the intensity of the green fluorescence signals, which represent the accumulation of intracellular reactive oxygen species (Cha et al., 2025).

### 2.6 Statistical analysis

All the quantitative data are presented as the means ± standard deviations (SDs). GraphPad Prism 9 was used for data plotting and statistical analysis. One-way analysis of variance (ANOVA) followed by Tukey’s multiple comparison test was performed for multigroup intergroup comparisons. Statistical significance was set at ^*^ *P* < 0.05, where ^*^ *P* < 0.05, ^**^ *P* < 0.01, and ^***^ *P* < 0.001 represent different levels of statistical significance; *ns* indicates no significant difference.

## 3 Results

### 3.1 Screening of shared therapeutic targets and hub genes

Venn diagram visualization of the overlapping relationships among the targets of AD-related genes, MCI-related genes, and gastrodin-predicted targets revealed a total of 127 intersecting genes, which were defined as the shared therapeutic targets of gastrodin for AD and MCI intervention (Figure 1A). The drug‒disease target regulatory network diagram intuitively reflects the multigene regulatory pattern by which gastrodin acts on AD and MCI pathological targets, demonstrating the simultaneous modulation of dozens of disease-associated functional genes by gastrodin (Figure 1B). A complete protein‒protein interaction network covering all 127 overlapping targets was constructed, and topological screening revealed multiple high-degree hub nodes, including ACHE, TNF, MAOA, PTGS2, SLC6A4, APP, ESR1, SERPINE1, TOP2A and S1PR5, consistent with the four core intersecting hub genes subsequently screened from clinical transcriptomic data (Figures 1C–E).

**FIGURE 1:**
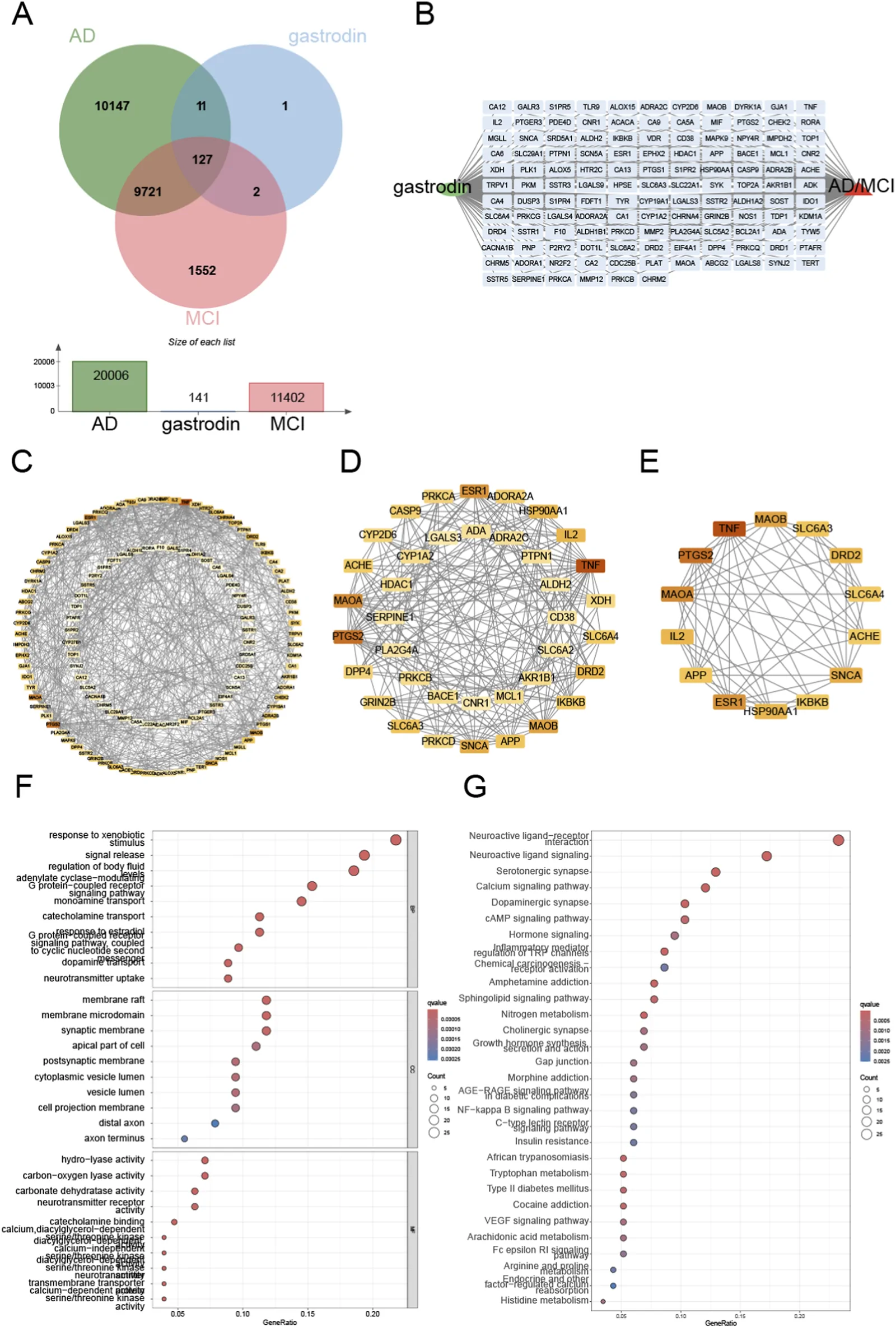
Network pharmacology analysis of the shared therapeutic targets of gastrodin in AD and MCI. **(A)** Venn diagram showing the overlapping relationships among AD-related targets, MCI-related targets, and predicted gastrodin targets. **(B)** Regulatory network of gastrodin and AD/MCI-associated target genes. **(C)** Complete protein‒protein interaction network constructed on the basis of shared therapeutic targets. **(D–E)** Core hub subnetwork stepwise screened by CytoNCA topological analysis. **(F)** Bubble plots of the Gene Ontology enrichment results for biological process, cellular component, and molecular function. **(G)** Bubble plots of significantly enriched Kyoto Encyclopedia of Genes and Genomes signaling pathways.

### 3.2 Functional enrichment analysis of therapeutic targets

Gene Ontology and Kyoto Encyclopedia of Genes and Genomes enrichment bubble plots revealed the core biological functions and signaling cascades enriched among the gastrointestinal therapeutic targets (Figures 1F,G). Biological process enrichment analysis revealed that the core functions were enriched primarily in the xenobiotic stimulus response, signal release regulation, and monoamine transmembrane transport. The dominant CC enrichment terms included membrane rafts, membrane microdomains, the synaptic membrane, the postsynaptic membrane, and the cell projection membrane. The enriched molecular function terms focused on hydrolyase catalytic activity, carbon‒oxygen lyase activity, and neurotransmitter receptor activity. The most significantly enriched Kyoto signaling pathways included neuroactive ligand‒receptor interactions, serotonergic and dopaminergic synapses, calcium signal transduction, cAMP signaling, NF‒κB neuroinflammatory pathways, and insulin resistance pathways. Taken together, these results indicate that gastrodin exerts anti-AD and anti-MCI neuroprotective effects mainly through regulating synaptic structural integrity, neurotransmitter signal transmission, intracellular calcium homeostasis and microglial inflammatory activation.

### 3.3 Identification and expression verification of core differential hub genes

Volcano plot visualization of differential gene screening results from the GSE329677 dataset captured 355 significantly upregulated and downregulated genes between AD patient samples and healthy control samples (Figure 2A). A cross-overlapping comparison of 355 differential genes related to AD and the therapeutic targets associated with gastrodin yielded only four intersecting core hub genes, namely, ACHE, S1PR5, SERPINE1 and TOP2A (Figure 2C). Box plot expression verification confirmed consistent significant differential expression of the four core genes across sample groups, with ACHE, S1PR5 and SERPINE1 exhibiting prominent upregulation in AD patient tissues, whereas TOP2A presented marked downregulation relative to healthy control samples, and all intergroup expression differences reached extremely significant statistical levels (Figure 2B).

**FIGURE 2:**
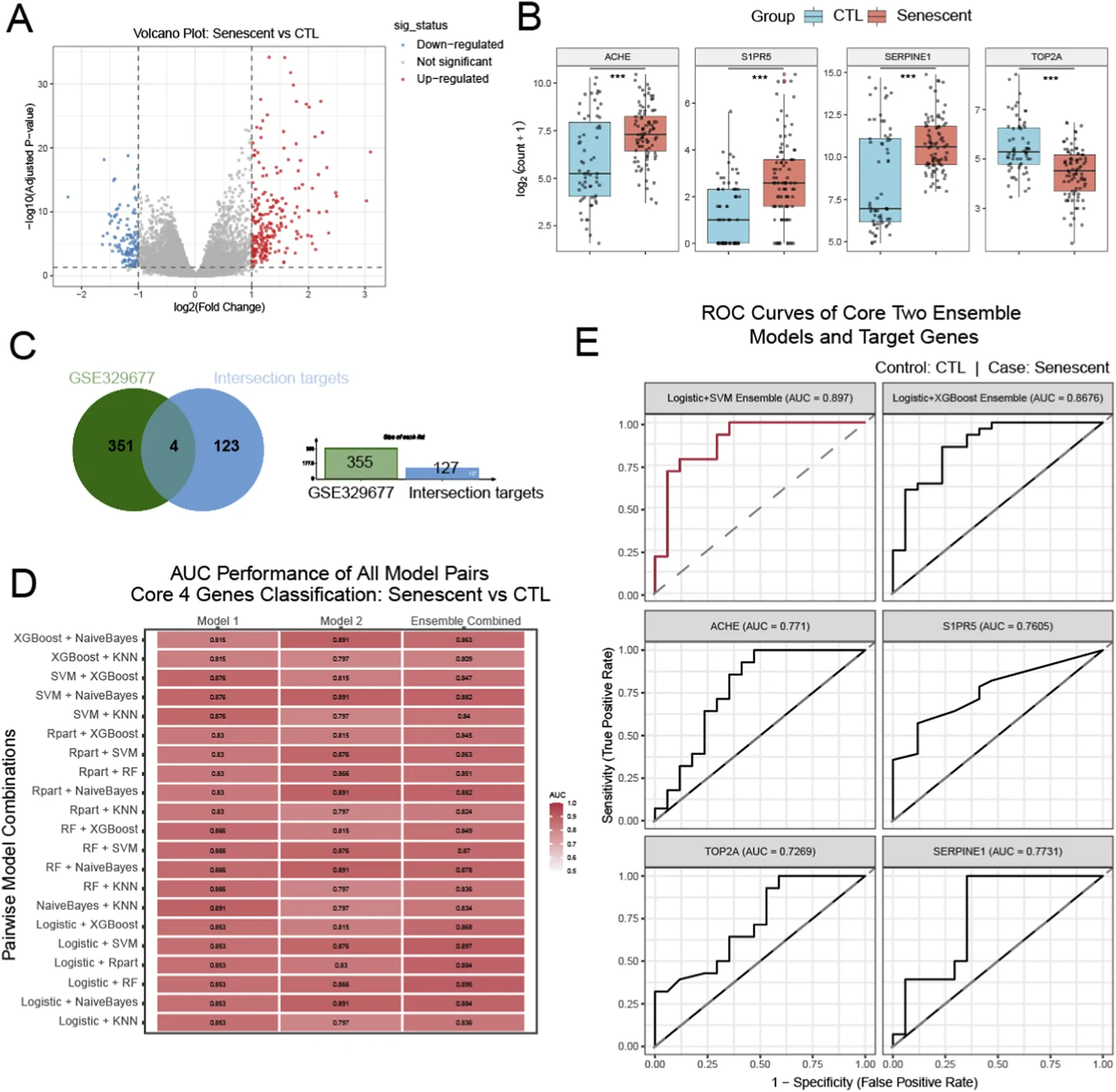
Transcriptomic differential gene screening and ensemble machine learning model construction based on the GSE329677 dataset. **(A)** Volcano plot of differentially expressed genes between senescent and control samples. **(B)** Box plots showing the differential expression levels of four core hub genes (*ACHE, S1PR5, SERPINE1, and TOP2A*) in different groups. **(C)** Venn diagram identifying four core intersecting hub genes from differentially expressed genes in AD patients and gastrointestinal therapeutic targets. **(D)** Heatmap summarizing the AUC values of all pairwise machine learning ensemble models. **(E)** ROC curves of the optimal ensemble models and individual ROC curves of the four core genes.

### 3.4 Diagnostic efficacy evaluation of core genes via machine learning

All pairwise ensemble machine learning classification models were constructed on the basis of the expression profiles of the four core genes. The heatmap of AUC values for all algorithm combinations revealed that the logistic regression-support vector machine (LR-SVM) ensemble model yielded the best diagnostic performance, with the highest AUC value of 0.897, followed by the logistic regression-random forest (LR-RF) model. The logistic regression-extreme gradient boosting (LR-XGBoost) model also exhibited favorable classification efficacy (Figure 2D). Receiver operating characteristic (ROC) curve comparison revealed that single core genes exhibited moderate diagnostic performance, with area under the curve values ranging from 0.7269 to 0.7731, whereas the ensemble model demonstrated comparatively good discriminatory power for distinguishing AD patients from healthy controls in this dataset, verifying that these four genes are core functional targets that mediate the therapeutic efficacy of gastrodin (Figure 2E).

### 3.5 Molecular docking analysis of gastrodin and core target proteins

Molecular docking confirmed favorable binding affinity between gastrodin and all four core target proteins, with gastrodin spontaneously embedding into the conserved ligand-binding pocket of each protein crystal structure (Figures 3A–D; Supplementary Table S1). Two-dimensional intermolecular interaction diagrams revealed abundant stable hydrogen bonds formed between gastrodin and key amino acid residues within each protein pocket. For the ACHE-gastrodin complex, hydrogen bond interactions formed with the TYR510, ARG525, VAL331 and VAL429 residues, with bond distances maintained between 1.9 and 3.2 Å. Gastrodin formed hydrogen bonds with the SER189, LEU318, MET101 and CYS148 residues within the S1PR5 binding pocket, whereas binding with SERPINE1 relied on hydrogen bond connections with LYS88, MET83, ILE91 and PRO85. Stable intermolecular hydrogen bonds between gastrodin and TOP2A were established with the PRO100, LYS251 and GLN97 residues. The short hydrogen bond distances across all the complexes reflected strong intermolecular binding forces, providing computational simulation-derived structural evidence for potential physical interactions between gastrodin and the four core target proteins; direct biochemical target-engagement assays (SPR or Co-IP) have not been performed. Re-docking validation showed that the RMSD values of ACHE, SERPINE1, and TOP2A were below 2.0 Å, indicating good reproducibility of native binding conformations. The RMSD of S1PR5 reached 2.855 Å, exceeding the threshold of 2.0 Å, which implied that the docking model for S1PR5 had limited ability to fully restore the crystal ligand pose, and the predicted binding modes should be interpreted cautiously (Supplementary Table S2).

**FIGURE 3:**
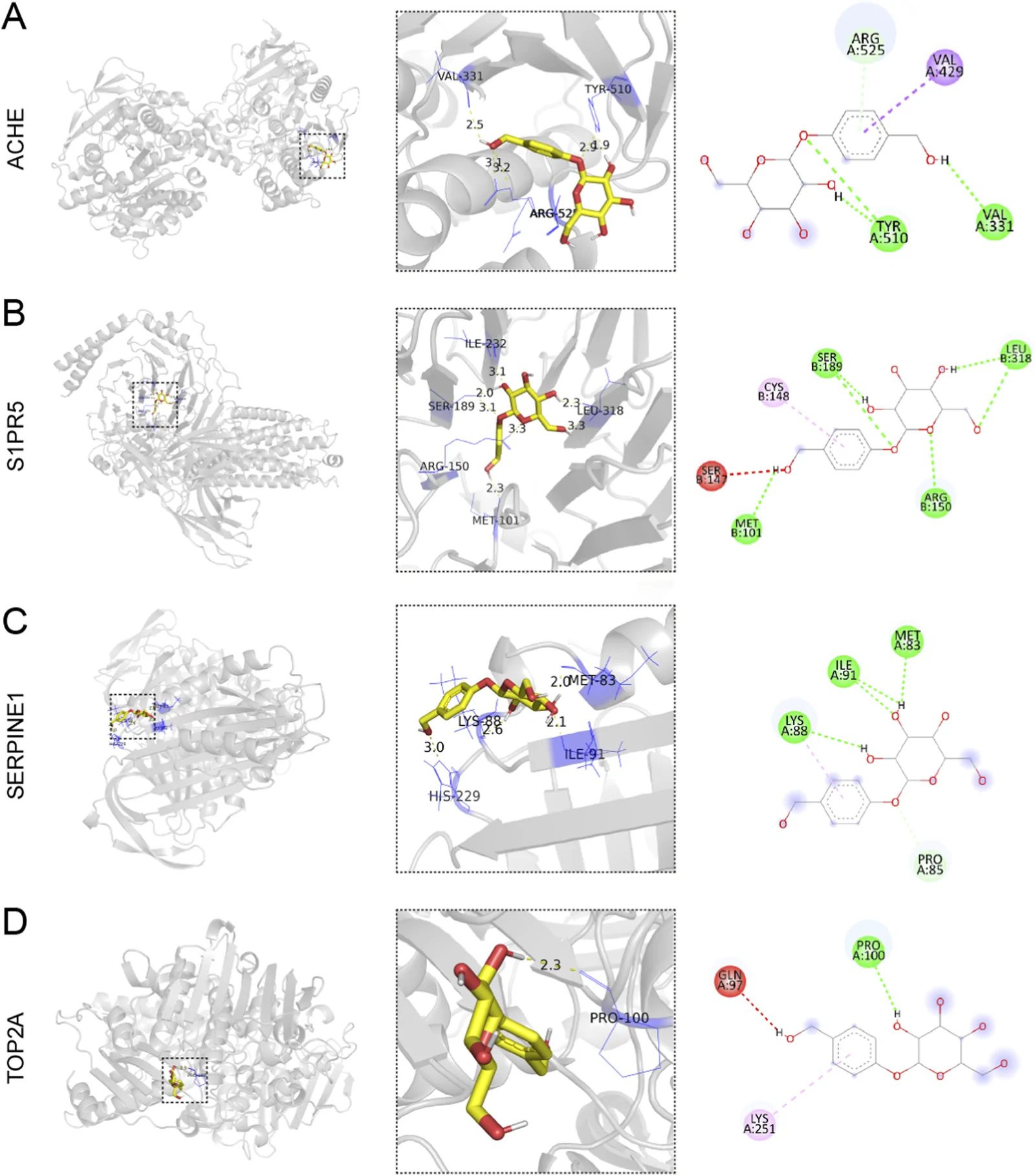
Molecular docking and intermolecular interaction analyses of the binding of gastrodin to four core target proteins. **(A–D)** Docking results of gastrodin with ACHE, S1PR5, SERPINE1, and TOP2A, respectively. Each panel presents the overall 3D docking conformation of the protein‒ligand complex, a magnified view of the ligand binding pocket with labeled hydrogen bond distances, and 2D diagrams displaying key amino acid residues involved in hydrogen bonding and hydrophobic interactions with gastrodin.

### 3.6 Verification of the complex stability via molecular dynamics simulation

Comprehensive trajectory analysis of the 50 ns molecular dynamics simulation revealed distinct stability characteristics among the four gastrodin–protein complexes. For gastrodin–S1PR5, gastrodin–SERPINE1, and gastrodin–TOP2A complexes, ligand and complex root-mean-square deviation curves (Figures 4A,B) reached equilibrium after ∼10 ns and fluctuated mildly without continuous upward drift, indicating that gastrodin remained stably anchored within the binding pocket. The gastrodin–ACHE complex was an exception; its complex RMSD rose gradually throughout the whole simulation without an obvious plateau, failing to achieve equilibrium within the 50 ns timescale.

**FIGURE 4:**
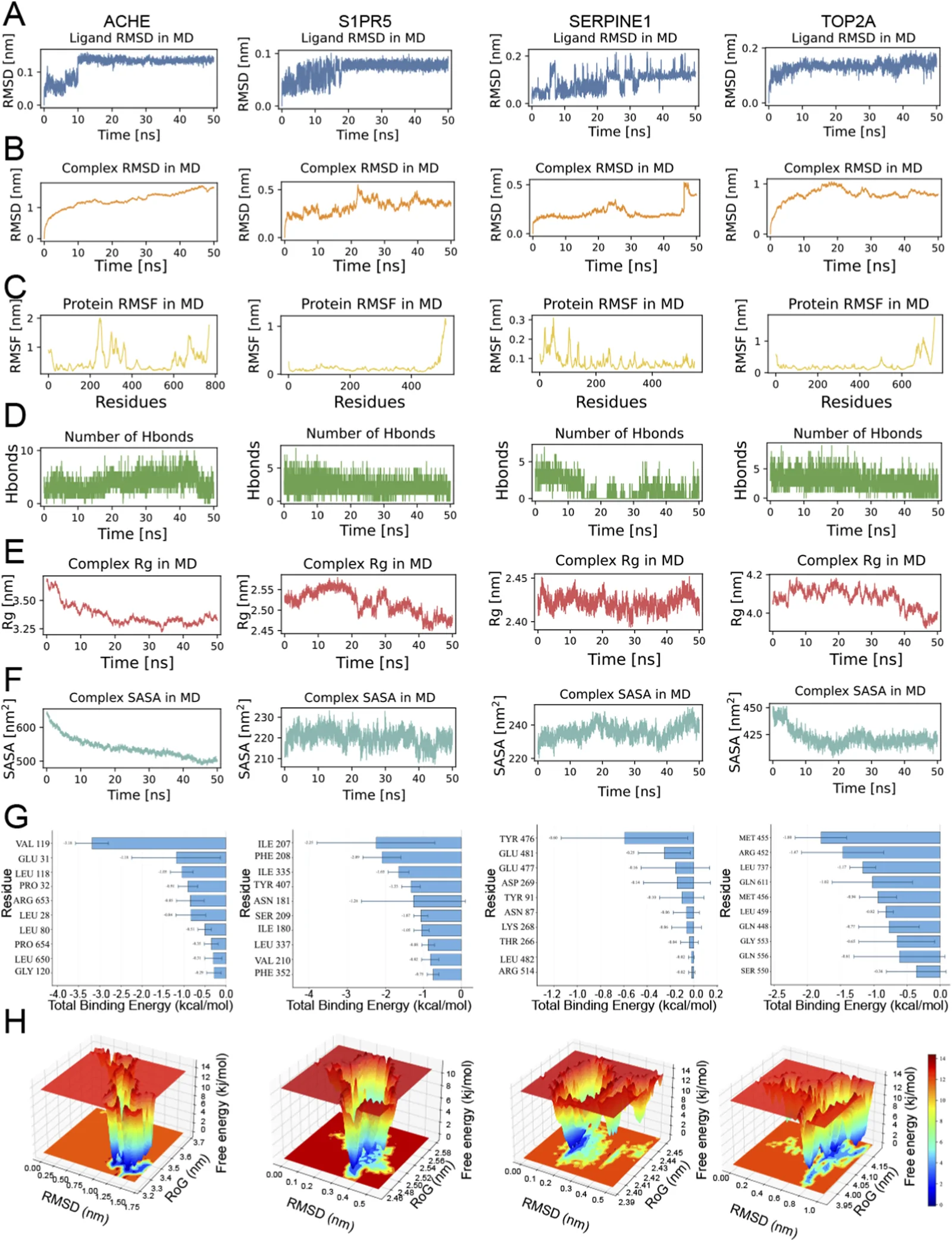
Trajectory analysis of 50 ns molecular dynamics simulations of four gastrodin-target complexes. **(A)** RMSD of the ligand. **(B)** RMSD of the whole protein–ligand complex; **(C)** RMSF of protein residues; **(D)** Number of persistent hydrogen bonds between gastrodin and each target. **(E)** Rg of the protein–ligand complex. **(F)** SASA of the protein–ligand complex. **(G)** Residue-level MM/GBSA binding energy decomposition; **(H)** FEL constructed using RMSD and Rg as collective variables to identify conformational energy minima.

The protein root-mean-square fluctuation curves (Figure 4C) demonstrated low flexibility at the ligand-binding region, suggesting gastrodin constrains conformational dynamics of the active domain. Continuous hydrogen bond quantity statistics (Figure 4D) showed persistent intermolecular interactions, with stable hydrogen bonds sustained throughout the simulation. Complex Rg (Figure 4E) fluctuated moderately after equilibration, indicating maintained structural compactness without protein unfolding. The SASA (Figure 4F) gradually declined and stabilized, implying gastrodin insertion reduces solvent exposure of the binding pocket.

Free energy landscapes (Figure 4H) exhibited a single deep energy minimum for each complex. Notably, FEL was plotted using RMSD and Rg as collective variables instead of PCA-derived principal components, consistent with MM/GBSA residue-level energy decomposition (Figure 4G) and total binding free energy results (Supplementary Table S3). These datasets only provide *in silico* computational evidence rather than biochemical experimental proof, supporting that the simulated docking pose represents a thermodynamically plausible binding conformation.

### 3.7 *In vitro* functional verification of the neuroprotective effects of gastrodin

*In vitro* functional assays systematically characterized the neuroprotective activity of gastrodin against Aβ oligomer-induced neuronal injury. The CCK-8 results revealed no statistically significant alteration in N2a-induced neuronal viability following treatment with gastrodin at concentrations ranging from 0.1 μM to 100 μM, confirming the biosafety of gastrodin within this concentration gradient and supporting the selection of 5 μM and 25 μM as intervention concentrations for subsequent functional experiments (Figure 5A). Quantitative analysis of propidium iodide (PI) immunofluorescence intensity revealed a markedly greater proportion of dead neurons in the Aβ oligomer-treated model group than in the control group (*P* < 0.001). Compared with the model group, the groups treated with both 5 μM and 25 μM gastrodin presented significantly reduced propidium iodide fluorescence signals reflecting neuronal death, with the 25 μM gastrodin group showing stronger antiapoptotic protective effects and highly significant intergroup differences (*P* < 0.001) (Figures 5B,D). DCFH-DA reactive oxygen species probe immunofluorescence imaging revealed extensive green fluorescent oxidative stress signals within neurons in the Aβ injury model group, whereas 25 μM gastrodin effectively suppressed the accumulation of intracellular reactive oxygen species (*P* < 0.01), directly demonstrating the protective effect of gastrodin against oxidative stress in damaged neurons (Figures 5C,E).

**FIGURE 5:**
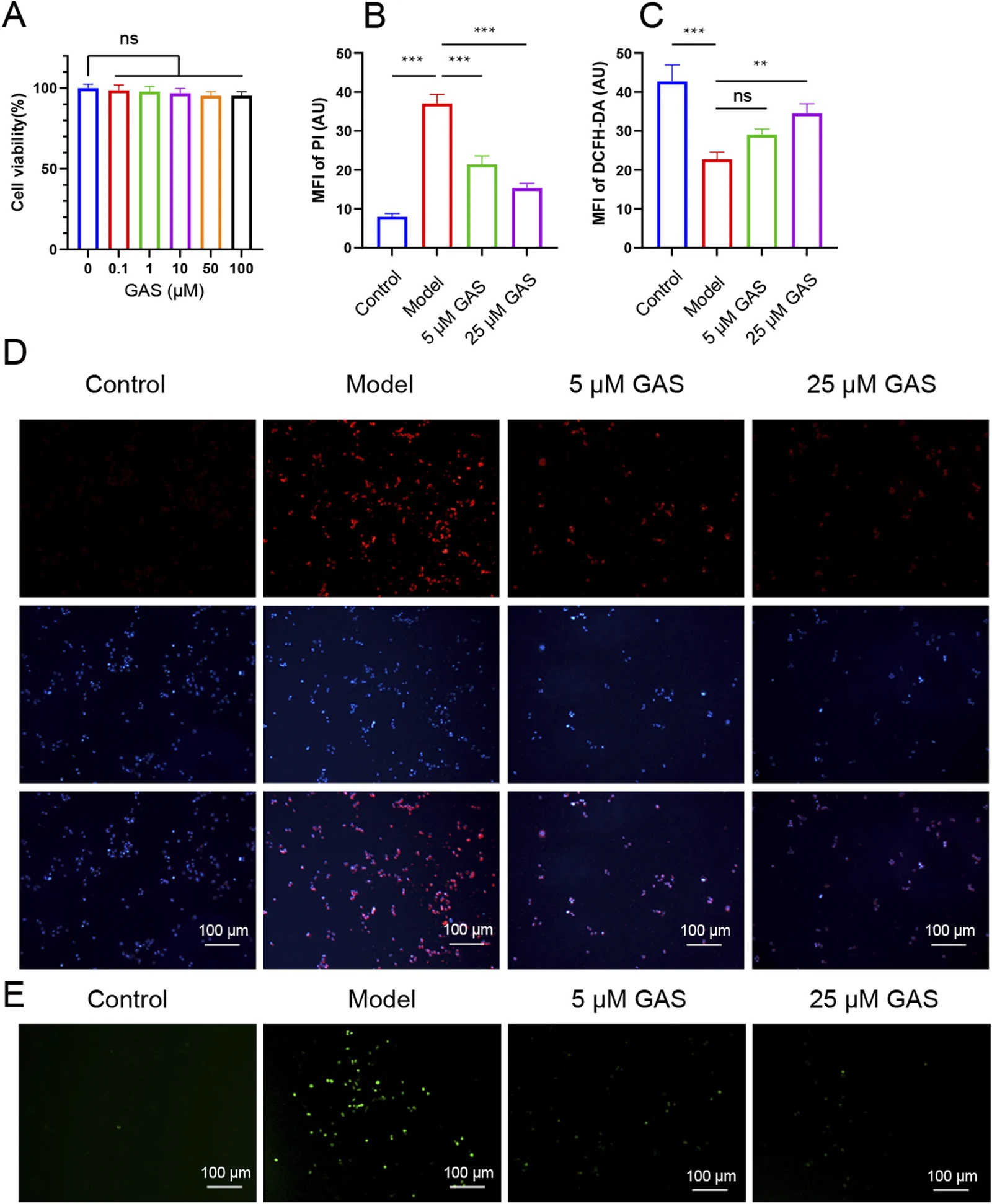
*In vitro* validation of the ability of gastrodin to protect against Aβ oligomer-induced neuronal injury in N2a neuronal cells. **(A)** CCK-8 assay for the viability of N2a neurons treated with serially diluted gastrodin (0–100 μM), *n* = 5. **(B)** Quantitative statistics of the mean fluorescence intensity from propidium iodide (PI) staining were used to evaluate the relative proportion of dead neurons across groups. **(C)** Quantification of the fluorescence intensity of intracellular reactive oxygen species (ROS) detected by the DCFH-DA fluorescent probe. **(D)** Representative micrographs of PI/Hoechst double staining showing neuronal apoptosis in each experimental group. **(E)** Representative DCFH-DA staining micrographs showing intracellular ROS accumulation in each group, *n* = 3. Scale bar = 100 μm **P < 0.01, ***P < 0.001 ns, no significant difference.

## 4 Discussion

### 4.1 Overview of the multitarget mechanism of gastrodin against AD/MCI

Alzheimer’s disease and its prodromal mild cognitive impairment are complex polygenic neurodegenerative disorders driven by multiple pathological cascades, including synaptic structural damage, Aβ plaque deposition, excessive oxidative stress and massive neuronal apoptosis (Kass et al., 2022). Gastrodin, a natural small-molecule neuroprotective monomer, exhibits broad-spectrum protective activity against central nervous system injury (Zheng et al., 2022); however, systematic identification of its core therapeutic targets and direct molecular binding verification remain insufficient. The present study constructed a complete layered research pipeline integrating network pharmacology omics prediction, transcriptome machine learning classification, computational structural simulation, and *in vitro* cellular functional validation to fully dissect the multitarget molecular mechanism by which gastrodin intervenes in AD and MCI pathological progression.

Network pharmacology target overlap analysis revealed 127 shared therapeutic targets of gastrodin that act on AD and MCI, and functional enrichment analysis revealed close correlations of these target genes with synaptic transmission, neurotransmitter receptor signaling, intracellular calcium homeostasis, and neuroinflammatory regulatory pathways. These enrichment results align with previously published reports confirming that gastrodin improves synaptic plasticity and inhibits microglia-mediated neuronal inflammatory injury (Huang et al., 2022; Zhang et al., 2024), whereas protein‒protein interaction network topological analysis preliminarily revealed multiple core hub genes, laying a foundation for subsequent clinical transcriptome-based core target filtering.

### 4.2 Identification and pathological significance of four core therapeutic targets

In this study, a hierarchical screening pipeline was applied to identify core targets of gastrodin against AD/MCI. First, 127 shared targets were obtained by intersecting gastrodin predicted targets and AD/MCI disease genes. Next, 355 AD differentially expressed genes were screened from the GSE329677 dataset (|log_2_FC| > 1, adjusted *P* < 0.05). Subsequent intersection analysis of these two gene sets, combined with PPI topological filtering and machine-learning feature selection, finally yielded four overlapping hub genes. We established three validation criteria: high topological centrality in the PPI network, favorable diagnostic AUC in integrated machine learning models, and verification in cellular experiments. This multi-stage filtering strategy reduces false positives and supports the reliability of ACHE, S1PR5, SERPINE1, and TOP2A as core functional targets.

To improve the clinical translational relevance of these predicted drug targets, in the present work, the human AD patient transcriptomic dataset GSE329677 was used to screen disease-related genes, which overoverlapped with the four core hub genes associated with the differential expression and drug regulatory potential of gastrodin. Acetylcholinesterase, encoded by ACHE, degrades the cholinergic neurotransmitter acetylcholine within synaptic clefts, and pathological overexpression of ACHE in AD brain tissue leads to severe cholinergic signal deficiency (Ferreira et al., 2021), representing the core pathological basis of learning and memory impairment (Mateev et al., 2023; Mehta et al., 2025). Cholinesterase inhibitors targeting ACHE currently constitute the first-line clinical symptomatic treatment for Alzheimer’s disease (Mehta et al., 2025). S1PR5 belongs to the sphingosine-1-phosphate receptor superfamily and regulates neuronal survival and blood–brain barrier integrity. Studies show diminished microglial S1PR5 promotes excessive microglial activation and neuroinflammation in AD (Czubowicz et al., 2026). However, the S1PR5 upregulation identified in our dataset originates from γ-irradiated senescent skin fibroblasts instead of AD brain tissue, and this discrepancy probably arises from cell-type heterogeneity. Preclinical findings reveal that S1PR5 activation improves cerebral sphingolipid disturbance and reduces Aβ burden, highlighting its therapeutic value for AD (Clausznitzer et al., 2018). SERPINE1 encodes plasminogen activator inhibitor-1 (PAI-1). Elevated SERPINE1 expression inhibits the activation of plasminogen mediated by tPA and uPA, thereby attenuating plasmin-dependent Aβ proteolysis. This impairment reduces cerebral parenchymal Aβ clearance efficiency and ultimately accelerates the deposition and aggregation of amyloid plaques in the brain, exacerbating AD pathological progression (Kutz et al., 2012; Picard et al., 2025). TOP2A encodes DNA topoisomerase IIα, which mediates neuronal DNA damage repair responses, and its abnormal downregulation facilitates neuronal cycle arrest and apoptosis under Aβ stimulation (Ouyang et al., 2026; Xie et al., 2025). The differential expression trend of the four core genes in AD patient samples fully illustrates their indispensable regulatory roles in multiple key pathological links of Alzheimer’s disease.

All pairwise ensemble machine learning classification models were constructed on the basis of expression profiles of the four core genes to quantify their diagnostic capacity for AD discrimination. The optimal logistic regression-support vector machine combined ensemble model achieved an area under the curve value of 0.897, substantially exceeding the moderate diagnostic efficiency of individual core genes (Zhang et al., 2026). This classification performance result not only suggests the combined detection of ACHE, S1PR5, SERPINE1 and TOP2A as potential peripheral blood diagnostic biomarkers for AD auxiliary screening but also confirms that these four genes constitute the core functional targets mediating the neuroprotective therapeutic effects of gastrodin.

### 4.3 Structural basis and functional verification of gastrodin neuroprotection

Combined molecular docking, 50 ns molecular dynamics simulation, and MM/GBSA binding free energy analysis form a three-tier *in silico* computational system to systematically interpret the atomic interaction mechanism between gastrodin and the four core targets ACHE, S1PR5, SERPINE1, and TOP2A. Static semiflexible molecular docking first visualized the binding mode of gastrodin spontaneously embedded into the conserved ligand pocket of each target protein (Bisht et al., 2024). Two-dimensional interaction diagrams identified abundant stable hydrogen bonds between gastrodin and pocket key residues, including TYR510/ARG525 of ACHE, SER189/LEU318 of S1PR5, LYS88/MET83 of SERPINE1, and PRO100/LYS251 of TOP2A. These docking results provided preliminary static computationally predicted structural evidence that gastrodin may potentially directly interact with the functional domains of the four disease-related hub proteins.

To compensate for the limitation of static docking snapshots, 50 ns all-atom MD simulations were performed to characterize the dynamic stability of gastrodin–protein complexes under physiological aqueous environments (Bai et al., 2023). Trajectory indicators including RMSD, root-mean-square fluctuation (RMSF), Rg and SASA revealed that gastrodin–S1PR5, gastrodin–SERPINE1 and gastrodin–TOP2A complexes reached equilibrium after ∼10 ns, whereas the gastrodin–ACHE complex failed to converge within the 50 ns simulation timescale. For the three equilibrated systems, gastrodin remained stably trapped in binding pockets without dissociation. Persistent intermolecular hydrogen bonds were sustained throughout the simulation, and low flexibility of ligand-binding domains further confirmed tight ligand anchoring. Based on the equilibrated trajectories, MM/GBSA calculations quantitatively evaluated binding affinity and residue-level energy contributions (Lin et al., 2026). Total binding free energy revealed that S1PR5 exhibited the strongest binding capacity to gastrodin, which matched the single deep energy minimum observed in free energy landscapes. Residue decomposition energies further validated that the hydrogen bond-forming residues identified by docking dominated favorable binding forces, building a closed-loop evidence chain from static interaction prediction to dynamic stability verification and quantitative affinity assessment. Collectively, these computational data *in silico* predict that gastrodin can durably occupy the functional pockets of core targets via stable hydrogen bond networks to regulate their catalytic or signal transduction activities; direct physical binding needs further biochemical validation.

An Aβ oligomer-induced N2a neuronal injury cellular model was adopted to validate the neuroprotective effect of gastrodin *in vitro*. CCK-8 viability detection confirmed the biological safety of gastrodin across the tested concentration range, while propidium iodide (PI) staining and reactive oxygen species (ROS) probe detection collectively demonstrated that gastrodin pretreatment significantly alleviated Aβ-triggered neuronal apoptosis and intracellular oxidative stress accumulation, with the optimal protective efficacy of 25 μM gastrodin. These neuroprotective effects are consistent with findings from previous relevant studies (Li et al., 2024; Tang et al., 2022). These cellular phenotypic results validated the neuroprotective activity of gastrodin predicted via prior computational omics analysis, which is consistent with the target regulatory mechanism inferred from molecular docking and dynamics simulation experiments.

### 4.4 Limitations and future perspectives

Several limitations of the present study require acknowledgment for objective discussion. This work involves only *in silico* predictive analysis and *in vitro* cellular functional verification and lacks *in vivo* AD transgenic animal model experiments to characterize regulatory changes in ACHE, S1PR5, SERPINE1 and TOP2A expression in brain tissue following systemic gastrodin administration. Second, direct biochemical target-engagement assays (e.g., surface plasmon resonance or co-immunoprecipitation) verifying the physical binding between gastrodin and these four candidate targets are absent; target binding is only inferred from computational predictions. Third, conformational entropy terms were omitted during MM/GBSA calculation; thus, only relative rather than absolute binding free energies can be interpreted from our simulation results. Fourth, we only counted the time-dependent variation of hydrogen-bond numbers across the simulation trajectories, while hydrogen-bond occupancy percentages for key interacting residues were not quantified, which limits our ability to further evaluate the persistence of hydrogen-bond interactions at individual key residue sites. Future work will employ APP/PS1 AD-transgenic mice to assess target expression changes upon gastrodin treatment. Biochemical binding assays and downstream pathway analyses will also be pursued in follow-up investigations.

## 5 Conclusion

In summary, this study systematically screened four core therapeutic targets of gastrodin against AD and MCI via a multidimensional integrated research framework, provided computational evidence of its potential structural binding through molecular docking and long-term molecular dynamics simulation, and validated the antiapoptotic and antioxidative neuroprotective effects of gastrodin in Aβ-damaged neurons. These findings clarify the multitarget molecular mechanism underlying gastrodin-mediated cognitive impairment intervention, identify novel candidate therapeutic targets, and provide a theoretical reference for the translational development of natural small-molecule anti-Alzheimer drugs.

## Data Availability

The datasets presented in this study can be found in online repositories. The names of the repository/repositories and accession number(s) can be found below: https://www.ncbi.nlm.nih.gov/, GSE329677.

## Glossary

Aβ: β-amyloid
AD: Alzheimer’s disease
ACHE: acetylcholinesterase
ANOVA: one-way analysis of variance
AUC: area under the curve
BP: biological process
CCK-8: cell counting kit-8
CC: cellular component
DCFH-DA: 2′,7′-dichlorodihydrofluorescein diacetate
DMEM: Dulbecco’s modified Eagle medium
FBS: fetal bovine serum
FEL: Free energy landscape
GAS: gastrodin
GO: Gene Ontology
KEGG: Kyoto Encyclopedia of Genes and Genomes
LPS: lipopolysaccharide
LR: logistic regression
MCI: mild cognitive impairment
MD: molecular dynamics
MF: molecular function
MM/GBSA: molecular mechanics/generalized born surface area
NF-κB: nuclear factor kappa-B
PBS: phosphate-buffered saline
PI: propidium iodide
PPI: protein-protein interaction
RMSD: root mean square deviation
RMSF: root mean square fluctuation
ROC: receiver operating characteristic
ROS: reactive oxygen species
SD: standard deviation
S1PR5: sphingosine-1-phosphate receptor 5
SERPINE1: serpin family E member 1
STAT3: signal transducer and activator of transcription 3
TLR4: toll-like receptor 4
TOP2A: topoisomerase IIα
TRAF6: TNF receptor associated factor 6
