# Supplementary material for: Gastrodin alleviates mild cognitive impairment and Alzheimer’s disease via candidate targeting of ACHE, S1PR5, SERPINE1, and TOP2A: an integrative computational and cellular study
Source: Front Pharmacol. 2026 Sep 29;17:1937359. doi: 10.3389/fphar.2026.1937359 (PMC13658492; doi:10.3389/fphar.2026.1937359)
Supplement: Supplementary file 1 [file Table1.docx]

Supplementary Material

# Supplementary Figures and Tables

**Table S1** Detailed molecular docking parameters used for AutoDock Vina

| Target protein | PDB ID | Grid center (x, y, z) (Å) | Grid size (x, y, z) (Å) | Spacing (Å) | Exhaustiveness | Number of docking modes | Energy range (kcal/mol) |
| --- | --- | --- | --- | --- | --- | --- | --- |
| ACHE | 4EY7 | -29.09, -35.051, 27.974 | 114.45,114.45,114.45 | 1.0 | 32 | 20 | 3.0 |
| S1PR5 | 7EW3 | 89.756,84.483,105.153 | 122..85,122..85,122..85 | 1.0 | 32 | 20 | 3.0 |
| SERPINE1 | 4D8O | -29.09,-35.051,27.974 | 126,126,126 | 1.0 | 32 | 20 | 3.0 |
| TOP2A | 1ZXM | -29.09,-35.051,27.974 | 126,126,126 | 1.0 | 32 | 20 | 3.0 |

**Table S2** Heavy-atom RMSD values derived from re-docking validation for each target protein

| Protein | PDB ID | RMSD (Å) |
| --- | --- | --- |
| ACHE | 7D9O | 1.441 |
| S1PR5 | 7EW1 | 2.855 |
| SERPINE1 | 3CVM | 0.872 |
| TOP2A | 1ZXM | 1.444 |

**Table S3** MM/GBSA binding free energy decomposition of gastrodin bound to ACHE, S1PR5, SERPINE1 and TOP2A (unit: kcal/mol)

| System name | ACHE | S1PR5 | SERPINE1 | TOP2A |
| --- | --- | --- | --- | --- |
| Δ*E*_vdw_ | -31.16±1.89 | -40.78±1.62 | -7.73± 6.06 | -31.85±1.33 |
| Δ*E*_elec_ | -28.03±1.94 | -21.79±5.58 | -13.14±11.42 | -21.88±6.08 |
| ΔG_GB_ | 46.53±2.70 | 41.42±4.66 | 19.81±14.09 | 38.22±6.31 |
| ΔG_SA_ | -5.02±0.17 | -5.77±0.14 | -1.42± 1.16 | -5.27±0.26 |
| ΔG_bind_ | -17.69±1.56 | -26.93±0.73 | -2.49± 3.44 | -20.78±3.27 |

Note: ΔE_vdW_: van der Waals energy. ΔE_elec_: electrostatic energy. ΔG_GB_: electrostatic contribution to solvation. ΔG_SA_: non-polar contribution to solvation. ΔG_bind_: binding free energy.
